# Prediction of spinal anesthesia-induced hypotension during cesarean delivery: a narrative review

**DOI:** 10.1186/s44158-026-00382-y

**Published:** 2026-04-11

**Authors:** Mina Adolf Helmy, Kerlous Adolf Helmy, Wael Mahmoud El Darandly, Haitham Hassan Awad, Fatma Morsy, Reham Amin Kaddah, Mohamed Ahmed Shamma, Lydia Magdy Milad

**Affiliations:** 1 Department of Anesthesia and Critical Care Medicine, Cairo University, Cairo, Egypt; 2 Department of Obstetrics, Gynecology and Perinatology, Sechenov University, Moscow, Russia; 3 Department of Anesthesia and Critical Care Medicine, Mataryah Teaching Hospital, Cairo, Egypt; 4 Anesthesia and Critical Care Medicine, Faculty of Medicine, Cairo University, Cairo, Egypt

**Keywords:** Spinal anesthesia-induced hypotension, Doppler indices, Velocity time integral

## Abstract

Spinal anesthesia-induced hypotension remains a major challenge in obstetric anesthesia, with significant maternal and fetal consequences despite prophylactic measures. Reliable prediction tools are essential to stratify risk and optimize management. This review narratively discusses evidence on clinical, hemodynamic, Doppler-derived, and pulse oximetry-based predictors of spinal anesthesia-induced hypotension in cesarean delivery. Clinical factors such as body mass index, maternal age, baseline blood pressure, and spinal anesthetic dose demonstrate variable predictive value. Hemodynamic markers, including heart rate variability and shock index, show promise but require further validation. Advanced monitoring techniques, such as stroke volume variation, subaortic velocity time integral, and femoral artery Doppler indices, exhibit high accuracy, while venous diameter-based measures (inferior vena cava, internal jugular vein, subclavian vein-axillary vein) yield inconsistent results. Pulse oximetry-derived indices, particularly the perfusion index, demonstrate moderate to good predictive ability across systematic reviews. Composite risk models integrating multiple parameters may enhance precision compared to single predictors. Overall, while several modalities show potential, translation into routine practice requires simplification, validation in larger cohorts, and integration into standard monitoring systems.

## Highlights

- Dynamic indices such as stroke volume variation, subaortic velocity time integral, and femoral artery Doppler demonstrate the strongest predictive accuracy for spinal anesthesia-induced hypotension compared to static venous measures.
- Pulse oximetry-derived indices, particularly the perfusion index, offer a practical, noninvasive tool with moderate to good predictive value, supported by systematic reviews.
- Composite models integrating clinical, hemodynamic, and Doppler parameters may enhance precision, but their incremental benefit over single dynamic predictors remains modest.

## Introduction

**Fig. 1:**
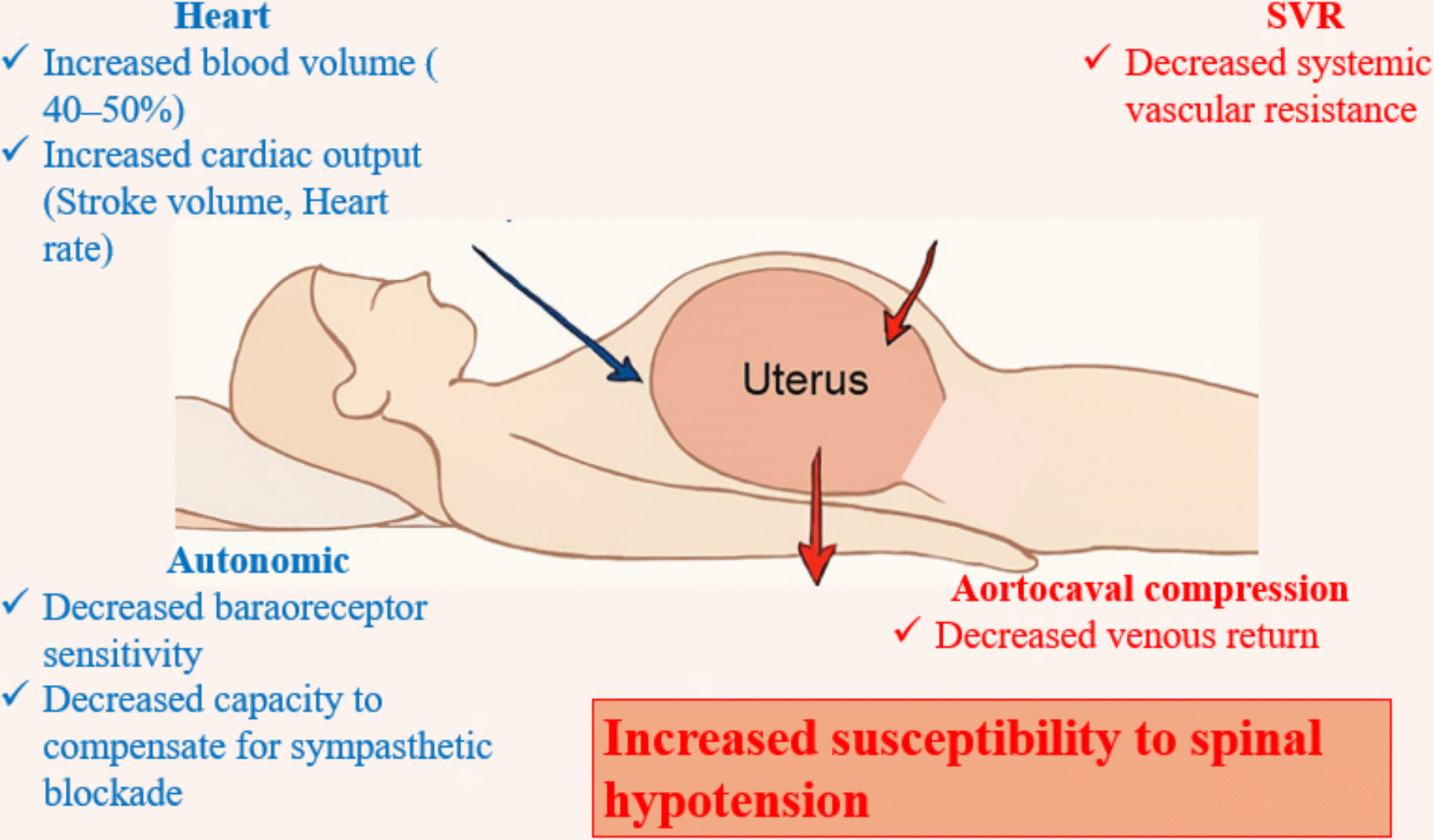
Maternal physiological changes predisposing to spinal anesthesia-induced hypotension

Spinal anesthesia-induced hypotension (SAIH) is a major concern in obstetric anesthesia [1, 2]. Pregnancy induces profound physiological adaptations that increase susceptibility to hypotension following spinal anesthesia. Blood volume expands by approximately 40–50%, accompanied by increased cardiac output due to enhanced stroke volume and heart rate. Systemic vascular resistance decreases under the influence of progesterone and prostacyclin, resulting in relative vasodilation. The gravid uterus exerts mechanical compression on the inferior vena cava and aorta, particularly in the supine position, which reduces venous return and cardiac preload. Autonomic regulation is also altered, with diminished baroreceptor sensitivity and reduced capacity to compensate for sympathetic blockade. Collectively, these changes predispose parturients to abrupt hypotension after spinal anesthesia [3] (Fig. 1).

Anesthetic management of SAIH centers on prevention and rapid intervention. Key strategies include positioning the patient with a left lateral tilt to reduce aortocaval compression, optimizing fluid administration through preloading or coloading, and using vasopressors such as phenylephrine or ephedrine to maintain maternal blood pressure. Continuous hemodynamic monitoring ensures early detection and timely treatment. Overall, management aims to stabilize maternal circulation while safeguarding fetal well-being [4].

Despite prophylactic fluid loading and vasopressor use, its incidence remains high. Hypotension can cause maternal discomfort, nausea, vomiting, and compromise uteroplacental perfusion, leading to fetal acidosis [4–6]. Reliable prediction methods would allow anesthesiologists to stratify risk and tailor interventions. This review examines available predictors, ranging from simple bedside markers to advanced monitoring technologies.

**Fig. 2:**
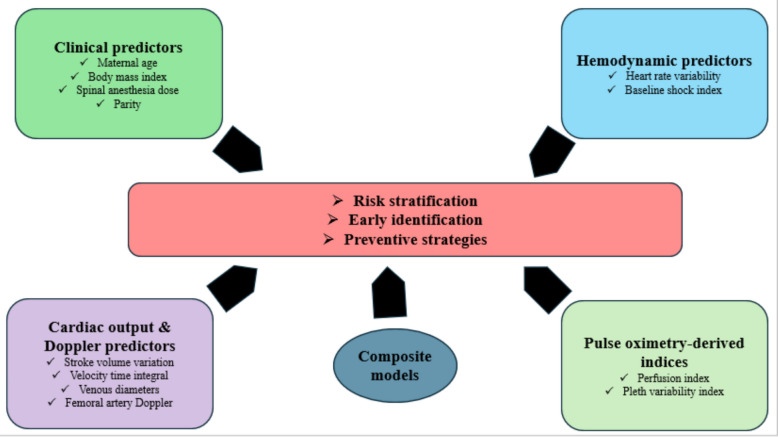
Overview of predictors of spinal anesthesia-induced hypotension

To facilitate a didactic presentation, this review is organized into clearly defined sections: clinical predictors, hemodynamic markers, cardiac output and Doppler indices, pulse oximetry-derived indices, and composite models (Table 1 and Fig. 2).

**Table 1:** Summary of predictors of spinal anesthesia-induced hypotension

Category	Predictor/Example	Strengths	Limitations
1. Clinical predictors	BMI, maternal age, baseline SBP, spinal dose [7–13]	Simple, bedside, widely available	Variable accuracy; confounded by patient heterogeneity; inconsistent evidence for multiparity/height
2. Hemodynamic predictors	HRV (Bishop et al. [14], LF/HF ratio ≥ 2.0)	Reflects autonomic control; physiologic insight	Complex methodology; limited clinical integration
3. Cardiac output & Doppler indices	SVV (Duclos et al. [17], cutoff 7%, AUROC 0.83)	Strong predictive accuracy; bedside applicability	Requires specialized device; limited availability
	Subaortic VTI (Zieleskiewicz et al. [18], ΔVTI ≥ 8%, AUROC 0.79)	Good predictive value; echocardiography-based	Operator-dependent; requires expertise/equipment
	IVCCI (Singh et al. [19])	Easy, non-invasive	Poor predictive accuracy; affected by positioning
	Femoral vein diameter (Yao et al. [20], RCFV > 12.2 mm)	A simple ultrasound measurement	Limited validation; moderate specificity
	IJVCI (Pharanitharan et al. [21], Elbadry et al. [22])	Easy bedside ultrasound	Conflicting evidence; poor sensitivity/specificity except at 38.5% cutoff
	SCV–AV Collapsibility (Asilantar et al. [23])	Non-invasive	No predictive value demonstrated
	Femoral Artery Doppler (Helmy et al. [16], ΔPI AUROC 0.99, ΔRI AUROC 0.96)	Very high accuracy; dynamic changes superior to baseline	Requires Doppler expertise; not widely available
4. Pulse oximetry-derived indices	Perfusion Index (PI), Pleth Variability Index (PVI) (AUROC ~ 0.75; PI sensitivity 0.81, specificity 0.75) [24, 25]	Non-invasive; widely available via pulse oximetry	Moderate accuracy; influenced by peripheral perfusion
5. Composite risk models	HR + LVEDA + VTI% (Feng et al. [26], AUROC 0.827)	Improved precision; integrates multiple parameters	More complex; not significantly better than VTI% alone

*SBP* Systolic blood pressure, *HRV* Heart rate variability, *LF/HF* Low-frequency to high-frequency ratiom, *SI* Shock Index, *SVV* Stroke volume variation, *VTI* Velocity time integral, *ΔVTI* Change in velocity time integral, *IVCCI* Inferior vena cava collapsibility Index, *IVCdmax/IVCdmin* Maximum/Minimum inferior vena cava diameter, *RCFV* Right common femoral vein, *IJV* Internal jugular vein, *IJVCI* Internal jugular vein collapsibility index, *SCV–AV* Subclavian vein–axillary vein, *PI* Pulsatility Index (Doppler)/Perfusion Index (pulse oximetry), *RI* Resistive Index, *PVI* Pleth variability index, *LVEDA* Left ventricular end-diastolic area, *AUROC* Area under the receiver operating characteristic curve, *CS* Cesarean section, *SAIH* Spinal anesthesia-induced hypotension

## Clinical predictors

Research findings demonstrated that body mass index and the dosage of spinal anesthesia are significant risk factors for hypotension during the induction of spinal anesthesia in cesarean sections [7, 8]. Additionally, advanced maternal age increases the risk due to reduced cardiovascular reserve. Lower pre-spinal systolic blood pressure is a predictor of more pronounced hypotension [7, 9]. Multiparity may influence autonomic responses, although current evidence remains inconsistent [7–10]. Height and vertebral column length also play a role, as taller women may experience greater cephalad spread of local anesthetic, leading to an enhanced sympathetic blockade [11–13].

## Hemodynamic predictors

### Heart rate variability (HRV)

Reduced HRV reflects impaired autonomic control and predicts hypotension. In this context, Bishop et al. [14] demonstrated that low-frequency-to-high-frequency (LF/HF) ratios can predict SAIH, with an optimal threshold of 2.0. HRV analysis techniques show considerable promise for predicting and managing obstetric spinal hypotension; however, their clinical application is limited by methodological complexity. There is an urgent need to simplify these techniques and integrate HRV analysis into conventional, commercially available monitoring systems. Such advancements would facilitate larger, pragmatic, and clinically relevant studies.

### Baseline shock index (SI)

Silwal et al. [15] demonstrated that a baseline SI of ≥ 0.9 was associated with SAIH in parturients undergoing emergency cesarean sections. In contrast, Helmy et al. [16] reported that an SI threshold of > 0.7 was predictive during elective cesarean sections. This discrepancy may be attributed to differences in the urgency of the procedure, with emergency cases potentially exerting greater physiological stress.

## Cardiac output and Doppler indices

### Stroke volume variation (SVV)

Elevated SVV indicates preload dependency and susceptibility to hypotension. Duclos et al [17] demonstrated that changes in SVV during passive leg raising, as measured by a digital non-invasive device, may serve as a useful predictor of hypotension. The area under the ROC curve for SVV in predicting hypotension was 0.83 (95% CI: 0.68–0.98; P = 0.001). An optimal SVV cutoff value of 7% was identified, corresponding to a sensitivity of 84% (95% CI: 0.70–0.99) and a specificity of 83% (95% CI: 0.69–0.97).

### Subaortic velocity time integral (VTI)

Zieleskiewicz et al. [18] demonstrated that variations in subaortic VTI, measured by echocardiography during leg raising, showed strong predictive value for SAIH. The area under the ROC curve for Δ VTI (semirecumbent wedged position) was 0.79 (95% CI: 0.63–0.90; p < 0.0001). The maximum Youden index was 0.53, and a threshold of 8% predicted hypotension onset with a sensitivity of 87%, specificity of 65%, positive predictive value of 58%, and negative predictive value of 87%.

### Venous diameters

#### Inferior vena cava collapsibility index (IVCCI)

Singh et al. [19] reported that the IVCCI is not a reliable predictor of SAIH in pregnant women undergoing elective cesarean section. They further demonstrated that the use of a wedge to achieve left lateral tilt increases the maximum inferior vena cava diameter (IVCdmax) without affecting the minimum diameter (IVCdmin) or the IVCCI.

#### Femoral vein diameter

Yao et al. [20] demonstrated that the transverse diameter of the right common femoral vein (RCFV), measured via ultrasound, was significantly associated with the occurrence of post-spinal hypotension during elective cesarean delivery. A transverse RCFV diameter greater than 12.2 mm was identified as a threshold predictive of major risk for hypotension following anesthesia in parturients undergoing cesarean section.

#### Internal jugular vein

Pharanitharan N. et al. [21] concluded that internal jugular vein (IJV) parameters, including maximum and minimum diameters as well as the IJV collapsibility index (IJVCI) measured preoperatively during spontaneous and deep breathing, are not reliable predictors of post-spinal hypotension in pregnant patients undergoing cesarean section. Although IJVCI is a simple and noninvasive method, it lacks predictive capability. These findings are consistent with those of Elbadry et al. [22], who reported poor sensitivity and specificity, with areas under the ROC curve of 0.55 (p = 0.377) during spontaneous breathing and 0.52 (p = 0.974) during deep breathing, corresponding to cut-off values of 29.5% and 37.5%, respectively. However, Elbadry et al. also found that a cut-off value of 38.5% for IJVCI was an efficient predictor of SAIH in pregnant patients.

#### Subclavian- axillary veins

Aslantar et al. [23] demonstrated that the preoperative subclavian vein–axillary vein (SCV-AV) collapsibility index is not a reliable predictor of SAIH in pregnant women undergoing elective cesarean section.

### Femoral artery Doppler

Helmy et al. [16] found that changes in femoral artery Doppler indices, specifically Doppler pulsatility index (PI), resistive index (RI), and waveform morphology, were highly accurate in predicting SAIH. Dynamic changes in PI and RI demonstrated greater predictive accuracy than baseline values. The area under the ROC curve (95% CI) was 0.99 (0.90–1.00) for ΔPI, 0.96 (0.85–1.00) for ΔRI, 0.77 (0.61–0.89) for baseline Doppler PI, and 0.68 (0.52–0.82) for baseline RI.

## Pulse oximetry-derived indices

A recent systematic review reported that baseline pulse oximetry perfusion index (PI) and pleth variability index (PVI) demonstrated moderate predictive ability for hypotension following spinal anesthesia in cesarean delivery, with an area under the summary ROC curve of approximately 0.75 for both indices [24]. Another systematic review found that the perfusion index exhibited good predictive accuracy, with pooled sensitivity and specificity values of 0.81 and 0.75, respectively [25]. Collectively, these findings suggest that the perfusion index may serve as a useful tool for predicting post-spinal hypotension.

## Composite models

Feng et al. demonstrated that combining heart rate (HR), left ventricular end-diastolic area (LVEDA), and velocity time integral percentage (VTI%) provided significantly better predictive accuracy for SAIH compared to HR alone (AUROC 0.827 vs. 0.707, p = 0.020) or LVEDA alone (AUROC 0.827 vs. 0.711, p = 0.039). However, the combined parameters were not significantly superior to VTI% alone (AUROC 0.827 vs. 0.766, p = 0.098). These findings suggest that integrating HR and LVEDA with VTI% may enhance precision in predicting hypotension compared to single-parameter assessment [26].

## Discussion

The prediction of SAIH in obstetric patients remains a complex challenge. This review highlights a wide spectrum of predictors, ranging from simple clinical variables to advanced hemodynamic and Doppler-derived indices. Clinical factors such as maternal age, BMI, and baseline blood pressure are easy to assess but lack precision due to inter-individual variability [11–13]. Hemodynamic markers such as HRV and the SI provide physiologic insight but are limited by methodological complexity and inconsistent thresholds across studies [14, 15].

Cardiac output and Doppler-based indices, particularly stroke volume variation, subaortic VTI, and femoral artery Doppler parameters, demonstrate strong predictive accuracy, suggesting that dynamic measures of preload and vascular resistance are more reliable than static venous collapsibility indices [16–18].

Indeed, venous parameters such as IVCCI, IJVCI, and SCV–AV collapsibility have shown poor reproducibility and limited clinical utility [21–23]. Conversely, pulse oximetry-derived indices such as the perfusion index offer a practical, noninvasive option with moderate predictive value, supported by systematic reviews [24, 25].

Composite models integrating multiple parameters (e.g., HR, LVEDA, VTI%) may enhance predictive precision, though their incremental benefit over single dynamic measures remains modest [26].

Overall, the evidence suggests that dynamic, physiologically responsive indices outperform static anatomical measures. However, translation into routine practice is hindered by equipment requirements, operator dependency, and lack of standardized cutoff values.

In addition to predictive indices, anesthetic management strategies remain central to mitigating SAIH. Standard approaches include prophylactic fluid loading, vasopressor administration (phenylephrine or ephedrine), and left lateral tilt positioning to reduce aortocaval compression. Understanding maternal physiology in pregnancy, particularly increased blood volume, reduced vascular resistance, and altered autonomic regulation, provides essential context for tailoring these interventions. Integrating predictive tools with established management strategies may enhance maternal comfort and fetal safety during cesarean delivery.

## Limitations of predictive indices

Despite their strong predictive accuracy, several limitations restrict the routine use of predictive indices. Dynamic Doppler‑based measures such as LVOT VTI and femoral artery Doppler, while physiologically robust, can be technically demanding and uncomfortable in pregnant women. These challenges are particularly pronounced in populations with elevated BMI, where image acquisition and positioning are more difficult. Operator dependency further complicates feasibility, limiting widespread adoption despite promising results in controlled studies.

Dynamic indices, including pulse oximetry‑derived perfusion index, stroke volume variation, and Doppler parameters, are also influenced by maternal positioning, degree of sympathectomy, and baseline fluid status. In full‑term women, these factors may alter measurements independent of hypotension risk, making reliance on single cutoff values potentially misleading. Monitoring trends over time, particularly in response to volume resuscitation, may provide more clinically relevant information than isolated measurements.

Finally, although femoral artery Doppler parameters such as ΔPI and ΔRI demonstrate exceptional predictive accuracy, their clinical application remains constrained by operator dependency. Reliable acquisition and interpretation require significant expertise and specialized training, which restricts their use compared with automated indices such as the perfusion index derived from pulse oximetry. These limitations highlight the need for standardized protocols, operator training, and integration of predictive indices into conventional monitoring platforms to ensure reproducibility and clinical feasibility.

## Future directions

Future research should focus on translating promising predictors of SAIH into practical clinical tools. Standardization of cutoff values for indices such as stroke volume variation, subaortic velocity time integral, and perfusion index is essential to improve reproducibility across diverse obstetric populations. Large-scale, multicenter trials are needed to validate these predictors and establish their generalizability. Simplification of complex methodologies, such as heart rate variability analysis, and integration of predictive indices into conventional anesthesia monitors would enhance feasibility and adoption in routine practice. Composite risk models that combine clinical, hemodynamic, and Doppler-derived parameters may offer superior accuracy, and the application of machine learning could further refine predictive algorithms. Ultimately, the goal is to develop pragmatic, noninvasive, and widely accessible monitoring strategies that allow anesthesiologists to stratify risk, tailor prophylactic interventions, and improve maternal and fetal outcomes during cesarean delivery under spinal anesthesia.

## Conclusion

Spinal anesthesia-induced hypotension in cesarean delivery remains a frequent and clinically significant complication. Current evidence highlights a spectrum of predictors, ranging from simple bedside indices to advanced echocardiographic and Doppler-based measures. Among these, dynamic parameters such as stroke volume variation, subaortic velocity time integral, and femoral artery Doppler indices demonstrate the strongest predictive accuracy, while venous collapsibility indices are less reliable. The perfusion index, derived from pulse oximetry, emerges as a practical and noninvasive tool with moderate predictive value. Composite models combining multiple hemodynamic variables may further improve risk stratification. Future research should focus on standardizing cutoff values, simplifying complex methodologies, and embedding predictive indices into conventional monitoring platforms to enable pragmatic, clinically relevant applications. Ultimately, reliable prediction of SAIH will allow anesthesiologists to tailor prophylactic strategies, minimize maternal discomfort, and safeguard fetal well-being.

## Data Availability

No datasets were generated or analysed during the current study.
